# Association of 14-day continuous glucose monitoring-derived postprandial hyperglycemic area with 6-month change in urinary albumin-to-creatinine ratio in patients with newly diagnosed type 2 diabetes mellitus

**DOI:** 10.3389/fendo.2026.1890463

**Published:** 2026-08-19

**Authors:** Tianchi Yu

**Affiliations:** Department of Endocrinology, Nanjing Drum Tower Hospital, Affiliated Hospital of Medical School, Nanjing University, Nanjing, Jiangsu, China

**Keywords:** albuminuria, continuous glucose monitoring, diabetic kidney disease, postprandial hyperglycemia, type 2 diabetes mellitus

## Abstract

**Background:**

Early kidney involvement may already be present at the diagnosis of type 2 diabetes mellitus (T2DM), whereas glycated hemoglobin A1c (HbA1c) and fasting plasma glucose do not fully characterize postprandial exposure. We evaluated the association of continuous glucose monitoring (CGM)-derived postprandial hyperglycemic area (PPHA) with 6-month change in urinary albumin-to-creatinine ratio (UACR) in adults with newly diagnosed T2DM.

**Methods:**

This single-center retrospective study consecutively screened 381 adults with newly diagnosed diabetes who underwent professional CGM from January 1, 2020, to December 31, 2024. After predefined exclusions, 297 participants with valid 14-day CGM and baseline and 6-month UACR measurements were analyzed. PPHA was the daily mean threshold-excess area above 10.0 mmol/L during 0–4 h after each main meal, calculated from valid monitoring days beginning on the first complete day after all components of the initial treatment regimen had been started. The primary outcome was ΔlnUACR [ln(6-month UACR + 1)-ln(baseline UACR + 1)]; exploratory UACR worsening was secondary. Multivariable regression, restricted cubic spline analyses, treatment-interaction analyses, and sensitivity analyses were performed.

**Results:**

UACR worsening occurred in 88 participants (29.6%). ΔlnUACR and the frequency of UACR worsening showed an increasing descriptive pattern across PPHA quartiles, with significant overall between-group differences (both *P* < 0.001). After adjustment for age, sex, body mass index, systolic blood pressure, HbA1c, fasting plasma glucose, estimated glomerular filtration rate, and baseline lnUACR, each 5 mmol·h·L^-^¹·d^-^¹ increase in PPHA was associated with a 0.08 higher ΔlnUACR (95% confidence interval [CI], 0.05-0.12; *P* < 0.001) and higher odds of UACR worsening (odds ratio [OR], 1.73; 95% CI, 1.27-2.36; *P* < 0.001). The association was approximately linear (*P* for nonlinearity=0.932), remained directionally consistent after simultaneous adjustment for recorded chronic comorbidities, and showed no statistically significant interaction with initial metformin, insulin, sodium-glucose cotransporter 2 inhibitor, or glucagon-like peptide-1 receptor agonist therapy (all interaction *P*>0.05).

**Conclusions:**

Higher CGM-derived PPHA was associated with greater 6-month change in UACR after adjustment for fasting plasma glucose and HbA1c. PPHA may complement conventional glycemic measures in early kidney-risk assessment; external validation is required.

## 1 Introduction

Type 2 diabetes mellitus (T2DM) and its chronic complications constitute a growing global disease burden, and diabetes-related kidney injury is an important cause of chronic kidney disease and kidney failure (1). The onset of T2DM is usually insidious, and some patients already have albuminuria or abnormal glomerular filtration function at first diagnosis; therefore, early identification of kidney-susceptible phenotypes has important clinical significance (2).

The urinary albumin-to-creatinine ratio (UACR) reflects injury to the glomerular filtration barrier and is associated with risks of cardiovascular events, kidney function decline, and death (3). Even when UACR is below the conventional albuminuria threshold, higher levels may still be accompanied by an increased risk of chronic kidney disease progression (4). Because UACR has substantial within-person biological variability, clinical determination of persistent albuminuria requires repeated testing (5).

Glycated hemoglobin A1c (HbA1c) reflects longer-term mean glycemia, and fasting plasma glucose reflects a relatively static fasting state; however, neither fully describes postprandial glucose peaks, duration, or within-day variability. Postprandial hyperglycemia is an important component of overall hyperglycemic burden and may differ substantially among patients with similar HbA1c levels (6).

Continuous glucose monitoring (CGM) can continuously record interstitial glucose and provide dynamic metrics such as mean glucose, time in range (TIR), time above range (TAR), and glycemic variability; these metrics are associated with diabetic microvascular complications (7). The use of CGM in diabetic kidney disease is expanding and may complement the limitations of HbA1c in characterizing dynamic glycemic exposure (8). Related consensus statements also emphasize its role in identifying hyperglycemia, hypoglycemia, and glycemic fluctuations (9).

Postprandial hyperglycemia may affect the albumin filtration barrier through oxidative stress, inflammatory responses, endothelial dysfunction, and changes in glomerular hemodynamics (10). Postprandial hyperglycemic area (PPHA) integrates both the magnitude and duration of glucose above a prespecified threshold and may quantify postprandial burden more completely than a single peak value. This study evaluated the association between 14-day CGM-derived daily mean PPHA and 6-month UACR change, and examined whether the association persisted after adjustment for baseline fasting plasma glucose and HbA1c, whether it was modified by initial glucose-lowering therapy, and whether it was affected by chronic complications and comorbidities present at diagnosis.

## 2 Materials and methods

### 2.1 Study design and participants

This was a single-center retrospective study. Through the electronic medical record system, adult patients who were first diagnosed with diabetes and underwent 14-day professional CGM in the Department of Endocrinology, Nanjing Drum Tower Hospital, Affiliated Hospital of Medical School, Nanjing University, from January 1, 2020, to December 31, 2024, were consecutively identified according to the date of first visit. The inclusion period covered 5 years, and the latest eligible 6-month follow-up date was July 30, 2025. The 381 patients constituted all consecutive cases in the predefined source cohort, and no secondary sampling was performed according to PPHA, baseline UACR, or the 6-month UACR outcome. After 84 patients were excluded according to predefined criteria, 297 patients were included in the primary analysis.

Newly diagnosed diabetes was defined as no previous diagnosis of diabetes and no previous glucose-lowering therapy before the diagnostic visit, with the current visit meeting any of the following criteria for the first time: (1) fasting plasma glucose after a fast of at least 8 h ≥7.0 mmol/L; (2) 2-h plasma glucose during a 75-g oral glucose tolerance test ≥11.1 mmol/L; (3) HbA1c ≥6.5% measured by an assay certified by the National Glycohemoglobin Standardization Program (NGSP) and traceable to the Diabetes Control and Complications Trial (DCCT) reference method; or (4) random plasma glucose ≥11.1 mmol/L in the presence of classic symptoms of hyperglycemia or a hyperglycemic crisis. In patients without classic symptoms of hyperglycemia or hyperglycemic crisis, confirmation was required by repeating the same abnormal test on another day or by another diagnostic test from the same or a different sample reaching the corresponding threshold. When results from different diagnostic tests were discordant, the test above the diagnostic threshold was repeated. T2DM classification was determined by endocrinologists on the basis of age at onset, body-weight phenotype, history of ketosis, C-peptide, family history, and clinical course; in patients with atypical clinical phenotypes, islet autoantibody results were further considered (11).

The inclusion criteria were as follows: (1) age ≥18 years; (2) newly diagnosed T2DM; (3) completion of 14-day CGM after the initial diagnosis with data meeting quality requirements; (4) availability of baseline and 6-month follow-up UACR; and (5) complete major clinical, biochemical, comorbidity, and medication data. The exclusion criteria were as follows: (1) type 1 diabetes, latent autoimmune diabetes in adults, gestational diabetes mellitus, monogenic diabetes, pancreatogenic diabetes, or other specific types of diabetes; (2) diabetic ketoacidosis, hyperosmolar hyperglycemic state, severe infection, acute cardiovascular or cerebrovascular event, or recent major surgery/trauma; (3) known primary or secondary glomerular disease, nephrotic syndrome, chronic kidney disease stage 4-5, or estimated glomerular filtration rate (eGFR) <30 mL·min^-^¹·1.73 m^-^²; (4) urinary tract infection, gross hematuria, menstrual contamination, or other urine sample conditions affecting UACR interpretation; (5) use of glucocorticoids, immunosuppressants, or other medications that substantially affect glucose metabolism or urinary albumin excretion; and (6) malignant tumor, pregnancy, severe hepatic insufficiency, or missing key data.

The study was approved by the Ethics Committee of Nanjing Drum Tower Hospital, Affiliated Hospital of Medical School, Nanjing University (approval number: 2026lyl012). All data were de-identified before analysis, and the ethics committee waived individual informed consent according to institutional regulations.

### 2.2 Clinical data, chronic complications, and treatment information

Age, sex, body mass index (BMI), systolic blood pressure, diastolic blood pressure, smoking history, history of hypertension, and history of dyslipidemia were extracted from the electronic medical record system. Laboratory variables included fasting plasma glucose (FPG), HbA1c, fasting insulin, fasting C-peptide, total cholesterol, triglycerides, high-density lipoprotein cholesterol (HDL-C), low-density lipoprotein cholesterol (LDL-C), serum uric acid, serum creatinine, and UACR. eGFR was calculated using the 2021 Chronic Kidney Disease Epidemiology Collaboration (CKD-EPI) creatinine equation without the race coefficient (12).

Chronic complications and comorbidities recorded at diagnosis included hypertension, dyslipidemia, stable ischemic heart disease, previous stroke or transient ischemic attack (TIA), peripheral arterial disease, diabetic retinopathy, and diabetic peripheral neuropathy. Hypertension and dyslipidemia were determined according to a previous diagnosis or records of corresponding long-term medication use. Previous atherosclerotic cardiovascular disease (ASCVD) was defined as any of stable ischemic heart disease, previous stroke/TIA, or peripheral arterial disease. Acute cardiovascular or cerebrovascular events were handled as exclusion criteria, whereas stable chronic diseases were retained and treated as potential confounders (13). Stable ischemic heart disease referred to previous myocardial infarction, coronary revascularization, or imaging-confirmed coronary atherosclerosis, with no acute coronary event within 3 months before enrollment. Diabetic retinopathy was determined according to fundus photography, ophthalmologic examination, or previous ophthalmologic diagnosis records. Diabetic peripheral neuropathy was determined according to typical symptoms and signs, 10-g monofilament or vibration perception testing, or specialist neurologic diagnosis records (14).

Initial glucose-lowering therapy was defined as the treatment category initiated from diabetes diagnosis to within 24 h after CGM placement and continued for at least 10 valid post-treatment CGM monitoring days, including metformin, insulin, sodium-glucose cotransporter 2 (SGLT2) inhibitors, and glucagon-like peptide-1 receptor agonists (GLP-1 RAs). Patients could receive multiple medication classes simultaneously. Dose adjustment during monitoring for safety reasons did not change the initial treatment classification. Angiotensin-converting enzyme inhibitors or angiotensin II receptor blockers (ACEI/ARBs) and statins were recorded within the same time window. PPHA was calculated beginning on the first complete monitoring day after all components of the initial regimen had been started. Two investigators independently extracted and cross-checked all variables, medication categories, and treatment initiation times; any discrepancies were adjudicated and finalized by a third investigator.

### 2.3 Continuous glucose monitoring and PPHA calculation

All patients wore a professional, factory-calibrated CGM sensor (FreeStyle Libre Pro; Abbott Diabetes Care, Alameda, CA, USA) within 72 h after the initial diagnosis; interstitial glucose was recorded every 15 min, and monitoring was planned for 14 d (15). Patients recorded the start times of three main meals each day, snacks, vigorous exercise, treatment of hypoglycemia, and medication adjustments. Patients were included only if the overall data capture rate was ≥70% and at least 10 valid post-treatment PPHA monitoring days were available. A valid PPHA monitoring day required that the initial regimen had been established, the start times of all three main meals were clear, and data completeness in each 0–4 h postprandial window was ≥80%. If the next meal or a definite snack started within 2 h after a meal, that meal was not included in PPHA; if it started at 2–4 h, the window was truncated before the subsequent intake. These meal-window and truncation rules were prespecified exposure-calculation rules for this study.

The primary exposure was 14-day CGM-derived daily mean PPHA. For each valid main meal after all components of the initial treatment regimen had been started, the trapezoidal method was used to calculate the threshold-excess area above 10.0 mmol/L during the 0–4 h postprandial period. The areas from the three main meals on each valid monitoring day were summed and then averaged across valid monitoring days, with units of mmol·h·L^-^¹·d^-^¹. The formula was PPHA = {Σ∫ max[G(t)-10.0, 0]dt}/D, where G(t) is the CGM glucose at the corresponding time point and D is the number of valid post-treatment PPHA monitoring days. The 14-day mean glucose, glucose management indicator (GMI), coefficient of variation (CV), TIR (3.9-10.0 mmol/L), TAR (>10.0 mmol/L), and time below range (TBR; <3.9 mmol/L) were calculated simultaneously.

### 2.4 UACR measurement and outcome definitions

Baseline and 6-month UACR were both measured using the first morning midstream urine sample. Urinary albumin was measured by immunoturbidimetry, urinary creatinine was measured enzymatically, and UACR was reported as mg/g on the same laboratory platform. The 6-month follow-up allowed a target-date window of ±30 d. When multiple eligible tests were available within the same window, the first morning urine result closest to the target date was used; if two eligible tests were performed on the same day, their mean was used. Results obtained during fever, infection, vigorous exercise, gross hematuria, or urine sample contamination were not included in the analysis (16).

UACR was measured in mg/g and was right-skewed; therefore, ln(UACR + 1) was calculated. The primary outcome was ΔlnUACR, defined as ln(6-month UACR + 1)-ln(baseline UACR + 1). The secondary exploratory outcome was UACR worsening, defined as a ≥30% increase in 6-month UACR from baseline or conversion from baseline <30 mg/g to 6-month UACR ≥30 mg/g. Baseline UACR ≥30 mg/g was defined as baseline albuminuria status. Persistent albuminuria was determined according to repeated elevation on interval testing (17). Stratification and clinical interpretation of UACR and eGFR followed recommendations for risk assessment in diabetic kidney disease (18).

### 2.5 Statistical analysis

Statistical analyses were performed using R 4.4.2. Distributions of continuous variables were assessed using histograms, Q-Q plots, and the Shapiro-Wilk test. Approximately normally distributed variables are expressed as mean ± standard deviation, and comparisons among four groups were performed using one-way analysis of variance. Skewed variables are expressed as median [interquartile range] and were compared using the Kruskal-Wallis test. Categorical variables are expressed as number (percentage) and were compared using the *χ²* test; when expected cell counts were insufficient, the Fisher-Freeman-Halton exact test was used. When PPHA was used to define quartile groups, it was displayed only descriptively, and no between-group inferential test was performed for that row.

Multivariable linear regression was constructed with ΔlnUACR as the continuous outcome, and multivariable logistic regression was constructed with UACR worsening as the binary outcome. PPHA was modeled per 5 mmol·h·L^-^¹·d^-^¹ increase and as quartiles; the two forms were not entered into the same model simultaneously. Model 1 was adjusted for age, sex, and BMI. Model 2 was further adjusted for systolic blood pressure, HbA1c, eGFR, and baseline lnUACR. Model 3 further included baseline FPG and was specified as the primary model. Model 4 added five indicator variables to model 3: hypertension, dyslipidemia, previous ASCVD, diabetic retinopathy, and peripheral neuropathy, as an expanded adjustment for chronic comorbidities. Variables were prespecified according to a clinical causal framework rather than selected according to univariable *P* values. Residual normality, homoscedasticity, and influential observations were evaluated for linear models; calibration and influential observations were evaluated for logistic models. Variance inflation factors were calculated for all models.

Spearman correlation analysis was used to evaluate the univariable correlation between PPHA and ΔlnUACR. Restricted cubic splines used four knots at the 5th, 35th, 65th, and 95th percentiles of PPHA, with the median of 7.8 mmol·h·L^-^¹·d^-^¹ as the reference, and used the model 3 covariate framework. Treatment effect modification analyses were based on model 3 and included the main effects of metformin, insulin, SGLT2 inhibitors, GLP-1 RAs, ACEI/ARBs, and statins, with one PPHA × glucose-lowering treatment product term added at a time. Only one interaction term was tested in each model. Stratified effects according to treatment use and interaction *P* values were reported, and the Benjamini-Hochberg method was used to control the false discovery rate (FDR) across eight interaction tests.

Sensitivity analyses included: (1) excluding participants with baseline UACR ≥30 mg/g; (2) excluding SGLT2 inhibitor or ACEI/ARB users; (3) standardizing PPHA per 1 standard deviation (SD; 9.6 mmol·h·L^-^¹·d^-^¹); (4) further adjusting for serum uric acid, triglycerides, and LDL-C; (5) obtaining robust standard errors for linear models using the heteroscedasticity-consistent covariance matrix estimator type 3 (HC3); and (6) applying Firth penalized likelihood regression to the primary continuous-PPHA logistic model. Major variables were complete among included patients, and multiple imputation was not performed. All tests were two-sided, and *P* < 0.05 was considered statistically significant.

No prospective recruitment target was set before clinical data were generated, and no *post hoc* power test was performed. All consecutive cases within the fixed 5-year source cohort were screened, and estimation precision was primarily evaluated by 95% confidence intervals (CIs) and model stability. The following parameter counts do not include intercepts: the primary continuous-PPHA logistic model included nine predictor parameters and 88 events, with approximately 9.8 events per parameter; the quartile model included 11 predictor parameters, with approximately 8.0 events per parameter. After five chronic comorbidities were added simultaneously in model 4, the continuous and quartile logistic models included 14 and 16 predictor parameters, respectively, with approximately 6.3 and 5.5 events per parameter. Therefore, model 4 was positioned as an exploratory extended model. Firth penalized logistic regression was used as an independent sensitivity analysis for the primary continuous-PPHA model and was not used to claim validation of model 4.

## 3 Results

### 3.1 Participant screening and baseline characteristics

**Figure 1:**
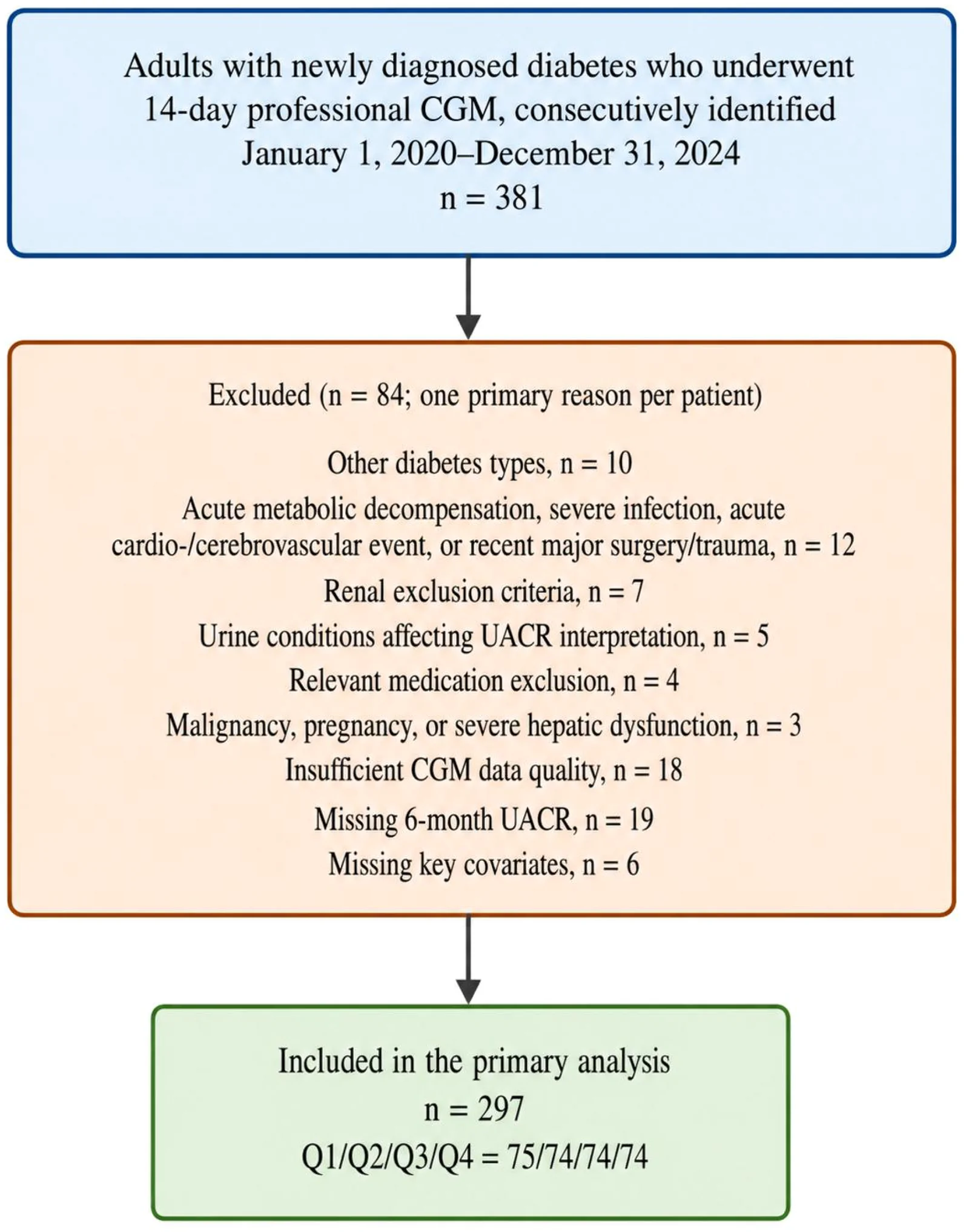
Flow diagram of participant screening. The source cohort comprised adults with newly diagnosed diabetes who underwent professional continuous glucose monitoring (CGM) and were consecutively identified from January 1, 2020, to December 31, 2024; the latest eligible 6-month follow-up date was July 30, 2025. UACR, urinary albumin-to-creatinine ratio; CKD, chronic kidney disease; eGFR, estimated glomerular filtration rate. Each patient was counted only according to the first primary exclusion reason.

From January 1, 2020, to December 31, 2024, 381 adults who were first diagnosed with diabetes and underwent 14-day professional CGM were consecutively identified, and no sampling according to exposure or outcome was performed within this source cohort. Ten patients with other types of diabetes were excluded, as were 12 with acute metabolic decompensation, severe infection, acute cardiovascular or cerebrovascular event, or recent major surgery/trauma; 7 with renal exclusion criteria; 5 with urine sample conditions affecting UACR interpretation; 4 because of relevant medication exclusion; 3 with malignancy, pregnancy, or severe hepatic insufficiency; 18 with insufficient CGM data quality; 19 with missing 6-month UACR; and 6 with missing key covariates. Each patient was counted only according to the first primary reason for exclusion. Ultimately, 297 patients were included in the primary analysis; the Q1, Q2, Q3, and Q4 groups included 75, 74, 74, and 74 patients, respectively (**Figure 1**).

Descriptively, age, systolic blood pressure, fasting plasma glucose, HbA1c, fasting C-peptide, total cholesterol, triglycerides, serum uric acid, and baseline UACR were higher or highest in Q4. Overall between-group differences for these continuous variables were statistically significant (all *P* < 0.05). The overall between-group distributions of male sex and history of hypertension also differed (both *P* < 0.05). No statistically significant overall between-group differences were observed for BMI, diastolic blood pressure, smoking history, history of dyslipidemia, previous ASCVD, stable ischemic heart disease, previous stroke/TIA, peripheral arterial disease, diabetic retinopathy, peripheral neuropathy, fasting insulin, HDL-C, LDL-C, serum creatinine, eGFR, or baseline albuminuria status (all *P*>0.05) (**Table 1**). No *post hoc* pairwise comparisons were performed; therefore, no inference regarding specific between-group significance was made.

**Table 1:** Baseline clinical, biochemical, and chronic complication characteristics stratified by postprandial hyperglycemic area (PPHA) quartiles.

Variable	Overall (n=297)	Q1 (n=75)	Q2 (n=74)	Q3 (n=74)	Q4 (n=74)	Statistic	*P* value
Age, years	51.5 ± 11.5	49.4 ± 10.2	49.8 ± 13.1	51.4 ± 11.4	55.4 ± 10.1	*F* = 4.39	0.005
Male	180 (60.6)	51 (68.0)	36 (48.6)	41 (55.4)	52 (70.3)	*χ²* = 9.88	0.020
BMI, kg/m²	25.3 ± 3.2	24.9 ± 3.0	25.2 ± 3.7	25.3 ± 2.9	25.7 ± 3.1	*F* = 0.80	0.495
Systolic blood pressure, mmHg	130.2 ± 13.1	125.9 ± 13.4	130.4 ± 12.5	130.1 ± 12.8	134.4 ± 12.8	*F* = 5.42	0.001
Diastolic blood pressure, mmHg	79.9 ± 8.3	79.6 ± 8.7	78.8 ± 8.3	79.2 ± 7.6	82.2 ± 8.2	*F* = 2.59	0.053
Smoking history	85 (28.6)	17 (22.7)	18 (24.3)	23 (31.1)	27 (36.5)	*χ²* = 4.43	0.219
History of hypertension	92 (31.0)	21 (28.0)	15 (20.3)	24 (32.4)	32 (43.2)	*χ²* = 9.56	0.023
History of dyslipidemia	149 (50.2)	39 (52.0)	35 (47.3)	32 (43.2)	43 (58.1)	*χ²* = 3.63	0.304
Previous ASCVD	28 (9.4)	5 (6.7)	4 (5.4)	7 (9.5)	12 (16.2)	*χ²* = 6.07	0.108
Stable ischemic heart disease	18 (6.1)	3 (4.0)	3 (4.1)	4 (5.4)	8 (10.8)	FFH	0.314
Previous stroke/TIA	8 (2.7)	1 (1.3)	1 (1.4)	2 (2.7)	4 (5.4)	FFH	0.462
Peripheral arterial disease	7 (2.4)	1 (1.3)	1 (1.4)	2 (2.7)	3 (4.1)	FFH	0.683
Diabetic retinopathy	24 (8.1)	3 (4.0)	4 (5.4)	6 (8.1)	11 (14.9)	*χ²* = 6.98	0.073
Diabetic peripheral neuropathy	35 (11.8)	5 (6.7)	7 (9.5)	8 (10.8)	15 (20.3)	*χ²* = 7.47	0.058
Fasting plasma glucose, mmol/L	8.3 ± 2.1	6.9 ± 1.2	7.4 ± 1.1	8.2 ± 1.2	10.6 ± 2.3	*F* = 85.24	<0.001
HbA1c, %	7.8 ± 1.3	7.1 ± 0.7	7.3 ± 0.9	7.8 ± 0.9	9.3 ± 1.4	*F* = 72.26	<0.001
Fasting insulin, mIU/L	10.8 [7.3,14.9]	9.4 [6.6,15.1]	10.5 [7.4,13.9]	10.2 [7.8,13.2]	12.3 [7.7,16.8]	*H* = 3.94	0.268
Fasting C-peptide, ng/mL	1.87 ± 0.55	1.82 ± 0.53	1.77 ± 0.51	1.81 ± 0.50	2.09 ± 0.58	*F* = 5.65	0.001
Total cholesterol, mmol/L	4.80 ± 0.82	4.46 ± 0.73	4.92 ± 0.84	4.79 ± 0.77	5.04 ± 0.84	*F* = 7.36	<0.001
Triglycerides, mmol/L	1.89 [1.35,2.78]	1.68 [1.16,2.42]	1.89 [1.31,2.80]	1.74 [1.30,2.61]	2.44 [1.68,3.68]	*H* = 19.03	<0.001
HDL-C, mmol/L	1.10 ± 0.25	1.09 ± 0.23	1.12 ± 0.26	1.12 ± 0.24	1.08 ± 0.26	*F* = 0.51	0.674
LDL-C, mmol/L	2.93 ± 0.70	2.83 ± 0.68	2.96 ± 0.67	2.86 ± 0.72	3.07 ± 0.72	*F* = 1.80	0.147
Serum uric acid, μmol/L	373.6 ± 83.4	359.6 ± 77.4	357.0 ± 83.3	392.2 ± 83.7	385.6 ± 84.9	*F* = 3.51	0.016
Serum creatinine, μmol/L	72.4 ± 12.4	74.6 ± 14.1	69.9 ± 12.4	72.1 ± 12.3	72.8 ± 10.5	*F* = 1.83	0.142
eGFR, mL·min^-^¹·1.73 m^-^²	98.9 ± 14.1	98.3 ± 15.0	101.7 ± 13.7	99.7 ± 13.2	95.7 ± 14.0	*F* = 2.40	0.068
Baseline UACR, mg/g	12.1 [7.0,21.9]	11.8 [6.9,17.9]	12.1 [7.3,17.4]	11.3 [5.7,21.2]	15.4 [9.2,27.4]	*H* = 9.36	0.025
Baseline UACR ≥30 mg/g	44 (14.8)	8 (10.7)	8 (10.8)	11 (14.9)	17 (23.0)	*χ²* = 5.87	0.118

PPHA, postprandial hyperglycemic area; Q1-Q4, PPHA quartiles from low to high; BMI, body mass index; HbA1c, glycated hemoglobin A1c; HDL-C, high-density lipoprotein cholesterol; LDL-C, low-density lipoprotein cholesterol; eGFR, estimated glomerular filtration rate; UACR, urinary albumin-to-creatinine ratio; ASCVD, atherosclerotic cardiovascular disease; TIA, transient ischemic attack; SD, standard deviation; IQR, interquartile range; n, sample size. Approximately normally distributed continuous variables are expressed as mean ± SD, skewed continuous variables as median [IQR], and categorical variables as n (%). *F* denotes the one-way analysis of variance statistic; *H* denotes the Kruskal-Wallis test statistic; *χ²* denotes the Pearson chi-square test statistic; FFH denotes the Fisher-Freeman-Halton exact test. *P* values are two-sided. ASCVD subtypes may overlap.

### 3.2 CGM metrics and initial treatment

No statistically significant overall between-group differences were observed in the number of valid PPHA monitoring days or data capture completeness (both *P*>0.05). The 14-day mean glucose, GMI, CV, and TAR were descriptively higher across higher PPHA quartiles, whereas TIR was descriptively lower, and overall between-group differences were statistically significant (all *P* < 0.001). No statistically significant overall between-group difference was observed for TBR (*P*>0.05). PPHA was the grouping variable and was displayed descriptively only. Among initial treatments, the overall between-group difference in insulin use was statistically significant, with the highest descriptive proportion in Q4 (*P* = 0.002). No statistically significant overall between-group differences were observed for metformin, SGLT2 inhibitors, GLP-1 RAs, ACEI/ARBs, or statins (all *P*>0.05) (**Table 2**).

**Table 2:** Continuous glucose monitoring (CGM) metrics and initial treatment stratified by postprandial hyperglycemic area (PPHA) quartiles.

Category	Metric	Overall (n=297)	Q1 (n=75)	Q2 (n=74)	Q3 (n=74)	Q4 (n=74)	Statistic; *P* value
CGM	Valid PPHA monitoring days, d	13.4 [13.0,13.8]	13.4 [13.1,13.8]	13.4 [12.9,13.8]	13.3 [13.0,13.7]	13.4 [13.0,13.7]	*H* = 0.87; 0.833
CGM	Data capture completeness, %	91.6 ± 5.0	91.7 ± 5.7	92.2 ± 4.8	90.9 ± 4.6	91.8 ± 4.7	*F* = 0.89; 0.448
CGM	PPHA, mmol·h·L^-^¹·d^-^¹	7.8 [4.0,13.0]	2.6 [2.1,3.4]	5.6 [4.8,6.3]	9.8 [8.9,11.1]	19.0 [15.6,29.8]	—
CGM	14-day mean glucose, mmol/L	9.2 ± 2.0	7.8 ± 0.9	8.2 ± 0.9	9.1 ± 0.9	11.7 ± 2.1	*F* = 133.62; <0.001
CGM	GMI, %	7.3 ± 0.9	6.6 ± 0.4	6.9 ± 0.4	7.3 ± 0.4	8.4 ± 0.9	*F* = 143.01; <0.001
CGM	CV, %	32.8 ± 6.4	30.7 ± 5.8	30.8 ± 5.7	32.6 ± 5.9	37.1 ± 6.2	*F* = 19.12; <0.001
CGM	TIR, %	57.3 ± 19.7	72.5 ± 7.7	68.0 ± 9.0	57.6 ± 8.2	31.1 ± 17.8	*F* = 195.15; <0.001
CGM	TAR, %	41.4 ± 19.7	26.5 ± 7.6	30.8 ± 9.5	40.9 ± 8.9	67.4 ± 18.0	*F* = 181.79; <0.001
CGM	TBR, %	1.0 [0.6,1.6]	1.0 [0.7,1.7]	1.0 [0.5,1.6]	1.2 [0.7,1.8]	1.1 [0.5,1.5]	*H* = 1.05; 0.790
Initial treatment	Metformin	236 (79.5)	61 (81.3)	56 (75.7)	61 (82.4)	58 (78.4)	*χ²* = 1.26; 0.738
Initial treatment	Insulin	91 (30.6)	17 (22.7)	19 (25.7)	19 (25.7)	36 (48.6)	*χ²* = 15.25; 0.002
Initial treatment	SGLT2 inhibitor	62 (20.9)	13 (17.3)	16 (21.6)	18 (24.3)	15 (20.3)	*χ²* = 1.14; 0.766
Initial treatment	GLP-1 RA	34 (11.4)	5 (6.7)	6 (8.1)	10 (13.5)	13 (17.6)	*χ²* = 5.55; 0.136
Initial treatment	ACEI/ARB	54 (18.2)	10 (13.3)	10 (13.5)	14 (18.9)	20 (27.0)	*χ²* = 6.19; 0.103
Initial treatment	Statins	146 (49.2)	37 (49.3)	32 (43.2)	37 (50.0)	40 (54.1)	*χ²* = 1.77; 0.622

CGM, continuous glucose monitoring; PPHA, postprandial hyperglycemic area; GMI, glucose management indicator; CV, coefficient of variation; TIR, time in range; TAR, time above range; TBR, time below range; SGLT2, sodium-glucose cotransporter 2; GLP-1 RA, glucagon-like peptide-1 receptor agonist; ACEI/ARB, angiotensin-converting enzyme inhibitor or angiotensin II receptor blocker; Q1-Q4, PPHA quartiles from low to high; SD, standard deviation; IQR, interquartile range; n, sample size. Approximately normally distributed variables are expressed as mean ± SD, skewed variables as median [IQR], and categorical variables as n (%). *F* denotes the one-way analysis of variance statistic; *H* denotes the Kruskal-Wallis test statistic; *χ²* denotes the Pearson chi-square test statistic; *P* values are two-sided. “—” indicates that no inferential test was performed because PPHA was used as the grouping variable. The target glucose range was 3.9-10.0 mmol/L. Initial glucose-lowering therapy was initiated from diagnosis to within 24 h after CGM placement and continued for at least 10 valid post-treatment CGM days; treatment categories could overlap.

### 3.3 PPHA groups and 6-month UACR change

**Figure 2:**
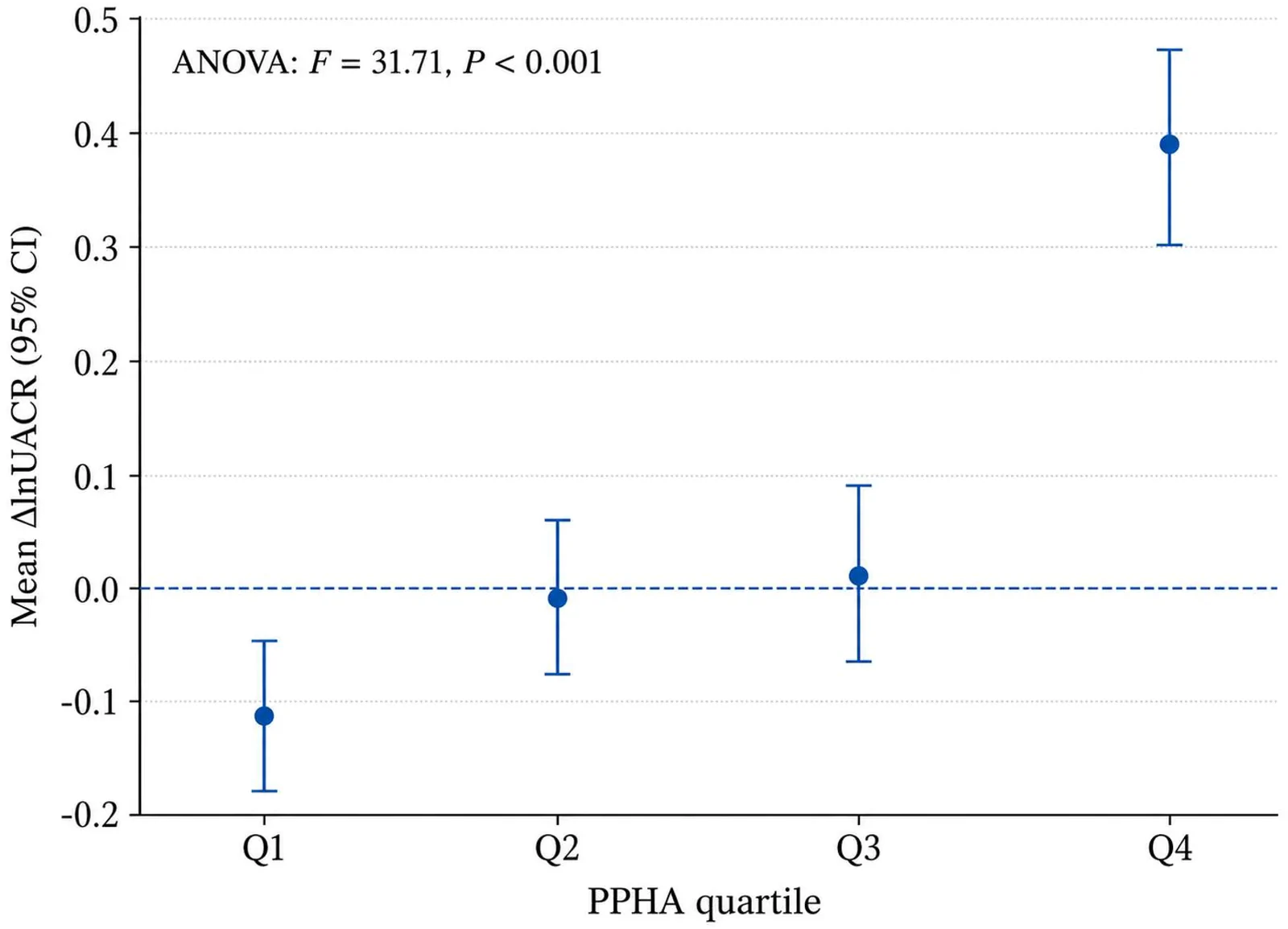
Mean ΔlnUACR and 95% CI stratified by postprandial hyperglycemic area (PPHA) quartiles. ΔlnUACR, ln(6-month urinary albumin-to-creatinine ratio [UACR]+1)-ln(baseline UACR + 1); CI, confidence interval; Q1-Q4, PPHA quartiles from low to high; ANOVA, analysis of variance. The Q1-Q4 cutoffs were ≤4.02, 4.03-7.81, 7.82-13.01, and >13.01 mmol·h·L^-^¹·d^-^¹, respectively. The group means and 95% CIs were -0.12 (-0.19 to -0.05), -0.01 (-0.08 to 0.06), 0.01 (-0.07 to 0.09), and 0.39 (0.30 to 0.48), respectively. Points and error bars represent group means and 95% CIs calculated from the *t* distribution. To avoid implying a continuous trend, quartile point estimates were not connected by lines. The overall between-group comparison used one-way analysis of variance, *F* = 31.71, *P* < 0.001.

**Figure 3:**
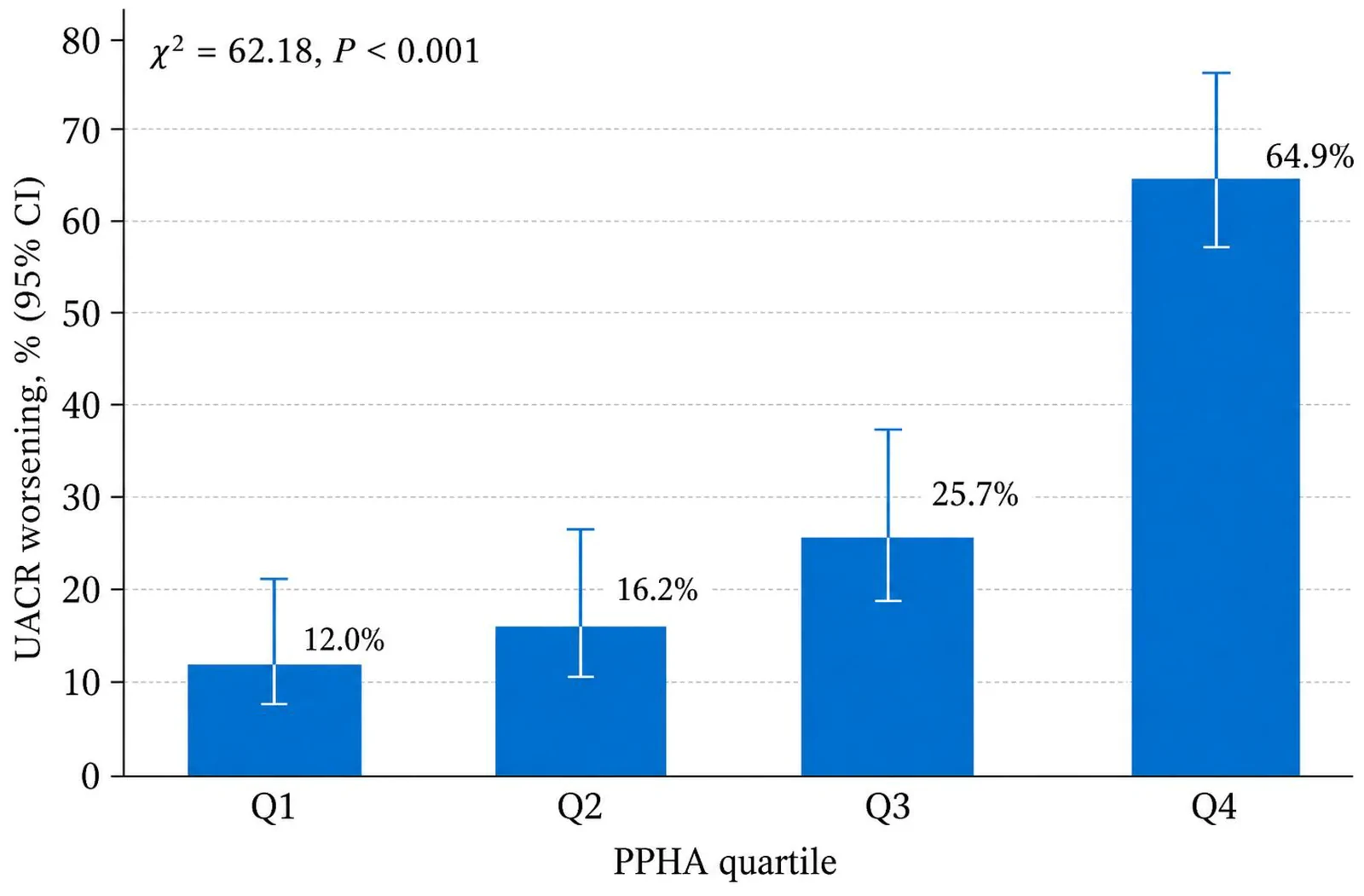
Proportion of UACR worsening and 95% CI stratified by postprandial hyperglycemic area (PPHA) quartiles. UACR, urinary albumin-to-creatinine ratio; CI, confidence interval; Q1-Q4, PPHA quartiles from low to high. Event counts were 9/75, 12/74, 19/74, and 48/74, corresponding to proportions and Wilson 95% CIs of 12.0% (6.4%-21.3%), 16.2% (9.5%-26.2%), 25.7% (17.1%-36.7%), and 64.9% (53.5%-74.8%), respectively. The overall between-group comparison used the Pearson *χ²* test, *χ²* = 62.18, *P* < 0.001.

Overall baseline UACR differed across PPHA quartiles, with a descriptively higher median in Q4 (*P* = 0.025). The 6-month UACR, absolute UACR change, and percentage change were descriptively highest in Q4, and overall between-group differences were statistically significant (all *P* < 0.001), although Q1-Q3 did not show a strictly monotonic pattern. Mean ΔlnUACR and the proportion with UACR worsening showed increasing descriptive distributions across PPHA quartiles, and overall between-group differences were statistically significant (both *P* < 0.001) (**Table 3**; **Figures 2**, **3**).

**Table 3:** Six-month urinary albumin-to-creatinine ratio (UACR) change stratified by postprandial hyperglycemic area (PPHA) quartiles.

Outcome	Overall (n=297)	Q1 (n=75)	Q2 (n=74)	Q3 (n=74)	Q4 (n=74)	Statistic	*P* value
Baseline UACR, mg/g	12.1 [7.0,21.9]	11.8 [6.9,17.9]	12.1 [7.3,17.4]	11.3 [5.7,21.2]	15.4 [9.2,27.4]	*H* = 9.36	0.025
6-month UACR, mg/g	12.8 [6.7,23.9]	10.5 [6.5,15.5]	10.7 [6.8,20.5]	10.4 [5.5,22.9]	23.9 [13.4,48.1]	*H* = 35.50	<0.001
ΔlnUACR	0.07 ± 0.39	-0.12 ± 0.29	-0.01 ± 0.31	0.01 ± 0.34	0.39 ± 0.41	*F* = 31.71	<0.001
Absolute UACR change, mg/g	0.4 [-2.3,4.4]	-0.8 [-3.3,0.9]	0.0 [-3.0,2.2]	-0.1 [-2.2,3.2]	6.6 [0.8,16.9]	*H* = 58.25	<0.001
Percentage UACR change, %	4.0 [-18.9,35.6]	-11.3 [-28.9,10.4]	0.2 [-23.1,22.1]	-0.7 [-23.8,29.1]	46.1 [10.1,96.3]	*H* = 62.62	<0.001
UACR worsening	88 (29.6)	9 (12.0)	12 (16.2)	19 (25.7)	48 (64.9)	*χ²* = 62.18	<0.001

PPHA, postprandial hyperglycemic area; UACR, urinary albumin-to-creatinine ratio; ln, natural logarithm; ΔlnUACR, ln(6-month UACR + 1)-ln(baseline UACR + 1); Q1-Q4, PPHA quartiles from low to high; SD, standard deviation; IQR, interquartile range; n, sample size. Approximately normally distributed variables are expressed as mean ± SD, skewed variables as median [IQR], and categorical variables as n (%). *F* denotes the one-way analysis of variance statistic; *H* denotes the Kruskal-Wallis test statistic; *χ²* denotes the Pearson chi-square test statistic; *P* values are two-sided. UACR worsening was defined as a ≥30% increase in 6-month UACR from baseline or conversion from baseline <30 mg/g to 6-month UACR ≥30 mg/g.

### 3.4 Correlation and dose-response relationship

**Figure 4:**
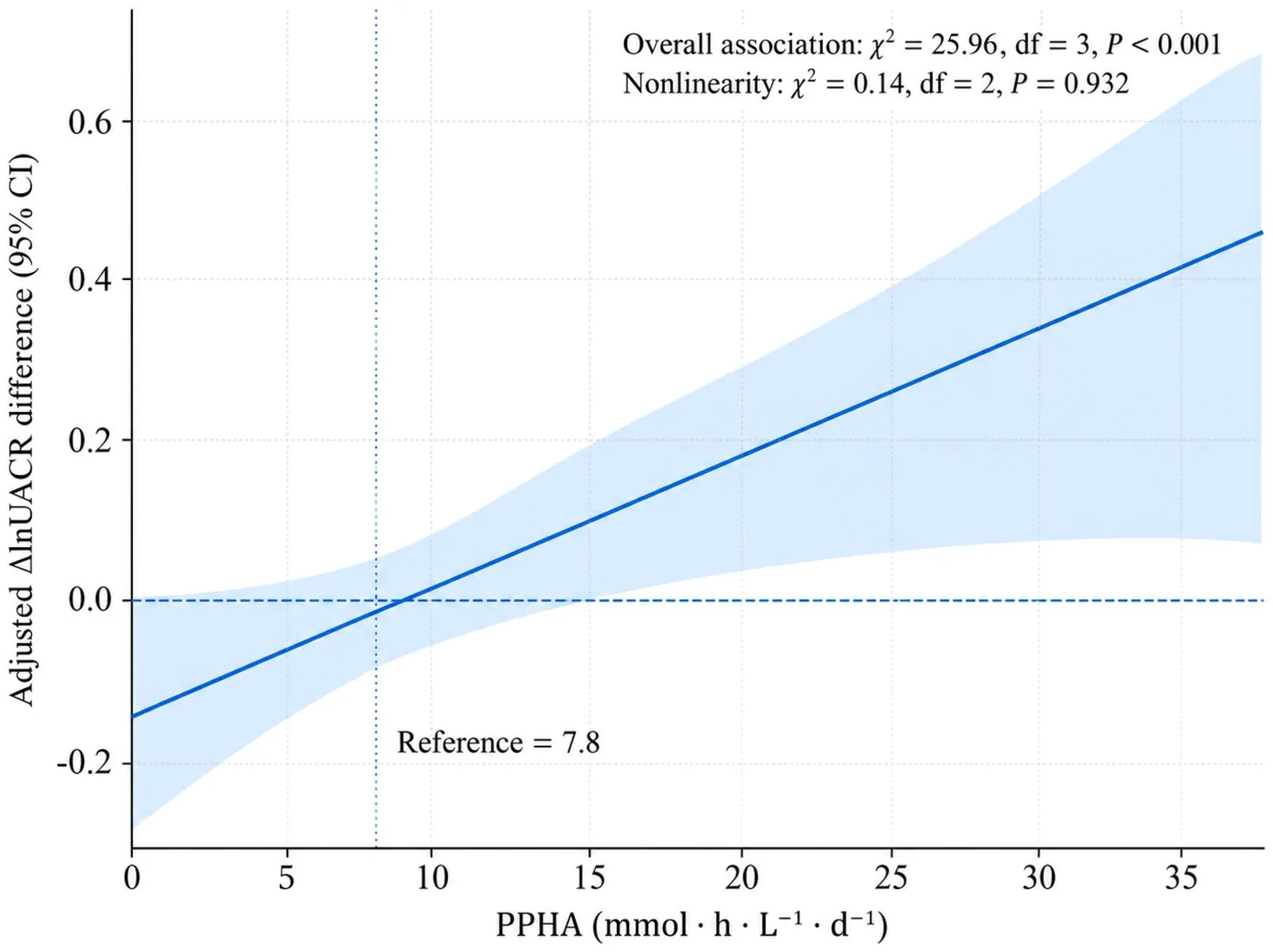
Restricted cubic spline curve for the association between postprandial hyperglycemic area (PPHA) and 6-month ΔlnUACR. ΔlnUACR, ln(6-month urinary albumin-to-creatinine ratio [UACR]+1)-ln(baseline UACR + 1); BMI, body mass index; HbA1c, glycated hemoglobin A1c; FPG, fasting plasma glucose; eGFR, estimated glomerular filtration rate; CI, confidence interval; df, degrees of freedom. The model was adjusted for age, sex, BMI, systolic blood pressure, HbA1c, FPG, eGFR, and baseline lnUACR. Knots were located at the 5th, 35th, 65th, and 95th percentiles of PPHA, with the median of 7.8 mmol·h·L^-^¹·d^-^¹ as the reference. The solid line represents the adjusted ΔlnUACR difference, the shaded area represents the 95% CI, the horizontal dashed line represents no difference, and the vertical dashed line represents the reference value. Overall association: *χ²* = 25.96, df = 3, *P* < 0.001; nonlinearity: *χ²* = 0.14, df = 2, *P* = 0.932.

PPHA was positively correlated with ΔlnUACR (Spearman *ρ* = 0.445, *P* < 0.001). In the restricted cubic spline model simultaneously adjusted for HbA1c and fasting plasma glucose, PPHA showed an increasing relationship with ΔlnUACR. The overall association was statistically significant (*χ²* = 25.96, df = 3, *P* < 0.001), whereas the test for nonlinearity was not statistically significant (*χ²* = 0.14, df = 2, *P* = 0.932), supporting an approximately linear association (**Figure 4**).

### 3.5 Multivariable linear regression

When PPHA was modeled as a continuous variable, it was associated with higher ΔlnUACR in models 1-4 (all *P* < 0.001). After FPG was included, the effect size was attenuated, and the direction and statistical significance of the association were maintained after simultaneous adjustment for five chronic comorbidities. In quartile models, the difference between Q4 and Q1 was statistically significant in all models (all *P ≤* 0.005), whereas the differences for Q2 and Q3 were not statistically significant in the primary and extended models (all *P*>0.05). The maximum variance inflation factor was <5 in all models (**Table 4**).

**Table 4:** Multivariable linear regression for the association between postprandial hyperglycemic area (PPHA) and 6-month ΔlnUACR.

PPHA modeling	Model	*β*	95% CI	*t* value	*P* value
Continuous: per 5 mmol·h·L^-^¹·d^-^¹ increase	Model 1	0.11	0.10-0.13	14.37	<0.001
Continuous: per 5 mmol·h·L^-^¹·d^-^¹ increase	Model 2	0.10	0.07-0.14	5.60	<0.001
Continuous: per 5 mmol·h·L^-^¹·d^-^¹ increase	Model 3 (primary)	0.08	0.05-0.12	4.88	<0.001
Continuous: per 5 mmol·h·L^-^¹·d^-^¹ increase	Model 4	0.08	0.04-0.12	4.18	<0.001
Q2 vs Q1	Model 1	0.10	-0.01-0.21	1.78	0.076
Q2 vs Q1	Model 2	0.07	-0.04-0.18	1.25	0.213
Q2 vs Q1	Model 3 (primary)	0.06	-0.05-0.17	1.09	0.277
Q2 vs Q1	Model 4	0.05	-0.06-0.17	0.90	0.370
Q3 vs Q1	Model 1	0.12	0.01-0.23	2.14	0.033
Q3 vs Q1	Model 2	0.05	-0.06-0.16	0.89	0.374
Q3 vs Q1	Model 3 (primary)	0.03	-0.08-0.15	0.59	0.558
Q3 vs Q1	Model 4	0.03	-0.09-0.15	0.49	0.624
Q4 vs Q1	Model 1	0.51	0.39-0.62	8.69	<0.001
Q4 vs Q1	Model 2	0.31	0.17-0.45	4.34	<0.001
Q4 vs Q1	Model 3 (primary)	0.24	0.09-0.39	3.17	0.002
Q4 vs Q1	Model 4	0.22	0.07-0.38	2.83	0.005

PPHA, postprandial hyperglycemic area; UACR, urinary albumin-to-creatinine ratio; ln, natural logarithm; ΔlnUACR, ln(6-month UACR + 1)-ln(baseline UACR + 1); Q1-Q4, PPHA quartiles from low to high; *β*, multivariable linear regression coefficient; CI, confidence interval; t, *t* test statistic for the regression coefficient; *P* values are two-sided; BMI, body mass index; HbA1c, glycated hemoglobin A1c; eGFR, estimated glomerular filtration rate; FPG, fasting plasma glucose; ASCVD, atherosclerotic cardiovascular disease. Model 1 was adjusted for age, sex, and BMI; model 2 was further adjusted for systolic blood pressure, HbA1c, eGFR, and baseline lnUACR; model 3 was further adjusted for FPG and was the primary model; model 4 added hypertension, dyslipidemia, previous ASCVD, diabetic retinopathy, and peripheral neuropathy to model 3. *β* and 95% CI are displayed to two decimal places; *t* values and *P* values are from unrounded model output and cannot be precisely recalculated from displayed values.

### 3.6 Multivariable logistic regression

When PPHA was modeled as a continuous variable, it was associated with higher odds of UACR worsening in models 1-4 (all *P ≤* 0.003). In quartile models, the odds were consistently higher for Q4 than Q1 (all *P ≤* 0.006), whereas the differences for Q2 and Q3 were not statistically significant in the primary and extended models (all *P*>0.05) (**Table 5**).

**Table 5:** Multivariable logistic regression for the association between postprandial hyperglycemic area (PPHA) and UACR worsening.

PPHA modeling	Model	OR	95% CI	Wald *χ²*	*P* value
Continuous: per 5 mmol·h·L^-^¹·d^-^¹ increase^-^¹	Model 1	1.94	1.58-2.37	41.05	<0.001
Continuous: per 5 mmol·h·L^-^¹·d^-^¹ increase	Model 2	1.99	1.49-2.66	21.66	<0.001
Continuous: per 5 mmol·h·L^-^¹·d^-^¹ increase	Model 3 (primary)	1.73	1.27-2.36	12.02	<0.001
Continuous: per 5 mmol·h·L^-^¹·d^-^¹ increase	Model 4	1.66	1.19-2.31	9.00	0.003
Q2 vs Q1	Model 1	1.35	0.52-3.49	0.38	0.537
Q2 vs Q1	Model 2	1.39	0.52-3.74	0.43	0.513
Q2 vs Q1	Model 3 (primary)	1.31	0.48-3.59	0.28	0.599
Q2 vs Q1	Model 4	1.27	0.44-3.67	0.20	0.655
Q3 vs Q1	Model 1	2.46	1.02-5.94	4.01	0.045
Q3 vs Q1	Model 2	2.11	0.82-5.43	2.40	0.122
Q3 vs Q1	Model 3 (primary)	1.85	0.69-4.97	1.49	0.222
Q3 vs Q1	Model 4	1.79	0.63-5.08	1.20	0.273
Q4 vs Q1	Model 1	12.80	5.31-30.85	32.26	<0.001
Q4 vs Q1	Model 2	8.21	2.82-23.94	14.89	<0.001
Q4 vs Q1	Model 3 (primary)	5.94	1.91-18.49	9.47	0.002
Q4 vs Q1	Model 4	5.52	1.65-18.46	7.69	0.006

PPHA, postprandial hyperglycemic area; UACR, urinary albumin-to-creatinine ratio; Q1-Q4, PPHA quartiles from low to high; OR, odds ratio; CI, confidence interval; Wald *χ²*, Wald test chi-square statistic; *P* values are two-sided; BMI, body mass index; HbA1c, glycated hemoglobin A1c; eGFR, estimated glomerular filtration rate; FPG, fasting plasma glucose; ASCVD, atherosclerotic cardiovascular disease. Model 1 was adjusted for age, sex, and BMI; model 2 was further adjusted for systolic blood pressure, HbA1c, eGFR, and baseline lnUACR; model 3 was further adjusted for FPG and was the primary model; model 4 added hypertension, dyslipidemia, previous ASCVD, diabetic retinopathy, and peripheral neuropathy to model 3. OR denotes the odds ratio for UACR worsening and is not equivalent to a risk ratio or a proportional increase in probability. ORs and 95% CIs are displayed to two decimal places; Wald *χ²* and *P* values are from unrounded model output and cannot be precisely recalculated from displayed values.

### 3.7 Effect modification by initial treatment

In prespecified interaction analyses, no statistically significant effect modification by metformin, insulin, SGLT2 inhibitors, or GLP-1 RAs was observed for the associations of PPHA with ΔlnUACR or UACR worsening. Interaction *P* values in linear models were 0.78, 0.73, 0.38, and 0.94, respectively, and interaction *P* values in logistic models were 0.86, 0.72, 0.54, and 0.81, respectively. The FDR-adjusted *q* values for all eight interaction tests were 0.94. The direction of subgroup effects was generally consistent, and the CIs were wider among SGLT2 inhibitor and GLP-1 RA users (**Supplementary Table 1**).

### 3.8 Sensitivity analyses

After excluding participants with baseline albuminuria, excluding SGLT2 inhibitor or ACEI/ARB users, standardizing PPHA, adding extended metabolic covariate adjustment, using HC3 robust standard errors, and applying Firth penalized likelihood regression to the primary continuous-PPHA logistic model, the direction and statistical significance of the associations of PPHA with ΔlnUACR and UACR worsening remained consistent. A total of 107 participants used SGLT2 inhibitors or ACEI/ARBs, including nine participants who used both medication classes, leaving 190 participants after exclusion (**Table 6**). The Firth analysis was an independent sensitivity analysis for the primary model and was not used to validate the more parameter-rich model 4.

**Table 6:** Sensitivity analyses for the associations of postprandial hyperglycemic area (PPHA) with UACR outcomes.

Analysis	n	PPHA scale	ΔlnUACR *β* (95% CI)	*t* value; *P* value	UACR worsening OR (95% CI)	Wald *χ²*; *P* value
Excluding baseline UACR ≥30 mg/g	253	Per 5 mmol·h·L^-^¹·d^-^¹ increase	0.08 (0.04-0.12)	3.92; <0.001	1.89 (1.31-2.73)	11.55; <0.001
Excluding SGLT2 inhibitor or ACEI/ARB users	190	Per 5 mmol·h·L^-^¹·d^-^¹ increase	0.07 (0.03-0.11)	3.43; 0.001	1.93 (1.28-2.92)	9.77; 0.002
Standardized PPHA	297	Per 1 SD (9.6 units)	0.15 (0.09-0.22)	4.72; <0.001	2.86 (1.58-5.20)	11.96; <0.001
Extended metabolic covariate adjustment	297	Per 5 mmol·h·L^-^¹·d^-^¹ increase	0.08 (0.05-0.12)	4.56; <0.001	1.71 (1.25-2.35)	11.18; 0.001
HC3 robust standard errors	297	Per 5 mmol·h·L^-^¹·d^-^¹ increase	0.08 (0.05-0.12)	4.61; <0.001	—	—
Firth penalized logistic regression	297	Per 5 mmol·h·L^-^¹·d^-^¹ increase	—	—	1.69 (1.26-2.28)	—; <0.001

PPHA, postprandial hyperglycemic area; UACR, urinary albumin-to-creatinine ratio; ln, natural logarithm; ΔlnUACR, ln(6-month UACR + 1)-ln(baseline UACR + 1); n, sample size; *β*, multivariable linear regression coefficient; OR, odds ratio; CI, confidence interval; t, *t* test statistic for the linear regression coefficient; Wald *χ²*, Wald test chi-square statistic; *P* values are two-sided; SD, standard deviation; LDL-C, low-density lipoprotein cholesterol; SGLT2, sodium-glucose cotransporter 2; ACEI/ARB, angiotensin-converting enzyme inhibitor or angiotensin II receptor blocker; HC3, heteroscedasticity-consistent covariance matrix estimator type 3. Firth penalized logistic regression denotes binary regression using penalized likelihood to reduce small-sample bias. “—” indicates not applicable or that the corresponding statistic was not reported. Unless otherwise specified, sensitivity analyses used the model 3 covariate framework; extended metabolic adjustment further included serum uric acid, triglycerides, and LDL-C. The *P* value in the Firth row was from the corresponding penalized likelihood model output. Effect estimates and 95% CIs are displayed to two decimal places; test statistics and *P* values are from unrounded model output.

## 4 Discussion

This study evaluated the relationship between 14-day CGM-derived PPHA and 6-month UACR change among consecutively screened patients with newly diagnosed T2DM. Higher PPHA was associated with greater ΔlnUACR and a higher proportion of UACR worsening. After simultaneous adjustment for HbA1c, fasting plasma glucose, and baseline kidney metrics, the association remained statistically significant, and the direction of results was consistent after hypertension, dyslipidemia, previous ASCVD, retinopathy, and peripheral neuropathy were additionally included. Restricted cubic splines supported an approximately linear relationship, and no statistically significant effect modification was observed for four classes of initial glucose-lowering therapy.

Newly diagnosed T2DM is often accompanied by insufficient early-phase insulin secretion, insulin resistance, and abnormal non-insulin-mediated glucose disposal, such that postprandial glucose exposure may differ among patients with similar mean glycemia (19). Under free-living conditions, dietary composition, postprandial activity, and sleep status may all affect postprandial glucose responses (20). Therefore, PPHA combines the magnitude and duration of above-threshold exposure into a single metric and more closely approximates cumulative postprandial hyperglycemic burden than a single postprandial peak.

Glycemic variability is associated with oxidative stress, inflammation, and multiple diabetic complications (21), and CGM-derived TIR has also shown consistent associations with the risk of microvascular complications (22). After model 2 was adjusted for HbA1c, the linear regression coefficient for continuous PPHA was 0.10 and the logistic regression OR was 1.99. After FPG was further included in model 3, the corresponding estimates decreased to 0.08 and 1.73. The attenuation of effect estimates suggests that fasting hyperglycemia explained part of the association; however, the residual multivariable-adjusted association may still be affected by unmeasured or inadequately measured confounding and should not be interpreted causally.

CGM may improve overall glycemic management in T2DM and provides within-day exposure information that cannot be shown by HbA1c (23). Evidence linking CGM-derived metrics to diabetic microvascular complications is accumulating (24). PPHA integrates the magnitude and duration of postprandial above-threshold exposure and provides a temporally structured description of dynamic glycemic exposure for short-term UACR change.

Postprandial hyperglycemia may affect urinary albumin excretion through interactions between immune-inflammatory and metabolic signaling, reactive oxygen species generation, and glomerular endothelial injury (25). Postprandial exercise can reduce postprandial glucose exposure (26), suggesting that PPHA may also be influenced by modifiable lifestyle factors.

Combination therapy addressing both basal and postprandial glycemia can improve CGM time-in-range metrics (27), and current pharmacologic treatment also emphasizes selection of glucose-lowering regimens according to hyperglycemic phenotype, hypoglycemia risk, and cardiorenal comorbidities (28). In this study, insulin use was more common in the high-PPHA group. After co-treatments, ACEI/ARB use, and statin use were simultaneously controlled, interaction tests for four classes of initial glucose-lowering therapy were not statistically significant, but CIs were wide among SGLT2 inhibitor and GLP-1 RA users.

SGLT2 inhibitors and renin-angiotensin system blockers can directly affect glomerular hemodynamics and albuminuria, and combined implementation of kidney-protective therapy is an important component of diabetes management in patients with chronic kidney disease (29). Results remained stable after users of these medications were excluded, reducing the likelihood that direct renal effects of these drugs fully explained the primary association. Hyperglycemic exposure may also maintain long-term tissue injury signals through metabolic memory (30).

Endothelial dysfunction connects postprandial glucose fluctuations, atherosclerosis, and changes in microvascular permeability (31). Covariate selection, overadjustment, and treatment-confounding feedback in observational data can substantially influence effect estimates (32). Therefore, this study used model 3 as the parsimonious primary model and included fasting plasma glucose as a key confounder. After hypertension, dyslipidemia, previous ASCVD, diabetic retinopathy, and peripheral neuropathy were added simultaneously in model 4, the directions of linear and logistic effect estimates remained consistent. However, this model included more predictor parameters and had a lower events-per-parameter ratio; therefore, it was used only as an exploratory extended adjustment. Retrospective determination of chronic complications also cannot exclude residual confounding due to missed diagnoses or misclassification.

Short-term within-person variability in UACR is high (33). Therefore, this study used continuous ΔlnUACR as the primary outcome and binary UACR worsening as an exploratory supportive outcome. The potential value of CGM in diabetic kidney disease management is increasing (34), but the repeatability, incremental predictive ability, and clinically interpretable range of PPHA still require external validation.

CGM use is associated with fewer diabetes-related hospitalization events (35), and poor long-term glycemic control is also associated with more rapid kidney function decline after the onset of diabetic kidney disease (36). The present findings support further evaluation of the repeatability and external validity of PPHA and its incremental predictive value beyond conventional risk models, but they do not directly justify setting intervention thresholds or selecting a specific treatment on this basis.

This study has several limitations. First, the single-center retrospective design cannot exclude residual confounding caused by unmeasured dietary composition, salt intake, physical activity, and medication adherence. Second, the source population included only patients with newly diagnosed diabetes who underwent professional CGM, and entry into the analysis additionally required eligible CGM and 6-month UACR data; therefore, selection bias related to clinical selection for CGM and follow-up completeness may have occurred. Consecutive screening was used within the 381-patient source cohort, and secondary sampling by PPHA or UACR outcomes was avoided, but bias caused by source-population restriction cannot be eliminated. Third, meal timing was based on patient diaries, and missed snacks or timing errors may have caused PPHA measurement error. Fourth, UACR has biological variability, and a single baseline and follow-up window cannot fully characterize persistent albuminuria. Fifth, some chronic complications were determined according to previous medical records, and asymptomatic disease not assessed by uniform screening may have been underestimated or misclassified. Sixth, the follow-up duration was only 6 months and could not evaluate sustained eGFR decline, kidney failure, or cardiovascular events. Seventh, treatment interaction analyses had limited sample size, treatment was not randomly assigned, and although FDR control was applied to multiple interaction tests, these results should be considered exploratory. Eighth, model 4 contained more parameters and had limited estimation precision; these results need validation in larger samples.

## 5 Conclusions

Among patients with newly diagnosed T2DM, 14-day CGM-derived daily mean PPHA was associated with increased 6-month ΔlnUACR and higher odds of UACR worsening. The association remained after simultaneous adjustment for HbA1c and fasting plasma glucose and maintained the same direction after recorded chronic comorbidities were simultaneously controlled. PPHA may serve as a supplementary dynamic exposure metric beyond conventional glycemic indices, but its external validity, incremental predictive value, and intervention implications require validation in multicenter prospective studies.

## Data Availability

The original contributions presented in the study are included in the article/**Supplementary Material**. Further inquiries can be directed to the corresponding author/s.
