# Supplementary material for: Association of 14-day continuous glucose monitoring-derived postprandial hyperglycemic area with 6-month change in urinary albumin-to-creatinine ratio in patients with newly diagnosed type 2 diabetes mellitus
Source: Front Endocrinol (Lausanne). 2026 Aug 19;17:1890463. doi: 10.3389/fendo.2026.1890463 (PMC13533769; doi:10.3389/fendo.2026.1890463)
Supplement: Supplementary file 1 [file Table1.docx]

**Supplementary Table 1. Effect modification by initial glucose-lowering therapy for the associations of postprandial hyperglycemic area (PPHA) with 6-month UACR outcomes.**

| **Treatment** | **Subgroup** | **n** | **ΔlnUACR *β* (95% CI)** | ***P* value** | **UACR worsening OR (95% CI)** | ***P* value** | **Interaction *P*: linear/logistic** |
| --- | --- | --- | --- | --- | --- | --- | --- |
| Metformin | Use | 236 | 0.08 (0.05-0.12) | <0.001 | 1.75 (1.24-2.47) | 0.001 | 0.78 / 0.86 |
| Metformin | Nonuse | 61 | 0.09 (0.03-0.15) | 0.003 | 1.86 (1.03-3.36) | 0.040 |  |
| Insulin | Use | 91 | 0.07 (0.03-0.12) | 0.003 | 1.63 (1.01-2.62) | 0.045 | 0.73 / 0.72 |
| Insulin | Nonuse | 206 | 0.08 (0.05-0.12) | <0.001 | 1.82 (1.27-2.61) | 0.001 |  |
| SGLT2 inhibitor | Use | 62 | 0.06 (0.00-0.12) | 0.050 | 1.49 (0.82-2.72) | 0.192 | 0.38 / 0.54 |
| SGLT2 inhibitor | Nonuse | 235 | 0.09 (0.06-0.12) | <0.001 | 1.85 (1.30-2.64) | <0.001 |  |
| GLP-1 RA | Use | 34 | 0.08 (-0.01-0.17) | 0.082 | 1.60 (0.69-3.74) | 0.276 | 0.94 / 0.81 |
| GLP-1 RA | Nonuse | 263 | 0.08 (0.05-0.11) | <0.001 | 1.79 (1.29-2.49) | <0.001 |  |

Note: PPHA, postprandial hyperglycemic area; UACR, urinary albumin-to-creatinine ratio; ln, natural logarithm; ΔlnUACR, ln(6-month UACR+1)-ln(baseline UACR+1); n, sample size; *β*, multivariable linear regression coefficient; OR, odds ratio; CI, confidence interval; *P* values are two-sided; interaction *P* values are Wald test results for the PPHA × treatment product term; FDR, false discovery rate; *q* value, *P* value adjusted by the Benjamini-Hochberg method; SGLT2, sodium-glucose cotransporter 2; GLP-1 RA, glucagon-like peptide-1 receptor agonist; ACEI/ARB, angiotensin-converting enzyme inhibitor or angiotensin II receptor blocker. Only one PPHA × glucose-lowering treatment interaction term was added at a time. The model included all model 3 baseline covariates, the main effects of the four classes of glucose-lowering therapy, ACEI/ARB, and statins. Treatment categories could overlap; the FDR-adjusted *q* values for all eight interaction tests were 0.94. Effect estimates and 95% CIs are displayed to two decimal places, and *P* values are from unrounded model output.
